# On the Statistics of the Mortality from Fractures of the Head

**Published:** 1850-08

**Authors:** 


*On the Statistics of the Mortality from Fractures of the Head.* By Dr. Fritze. The following statistical statements are founded upon the careful observation of 301 cases, by Dr. Fritze, of Nassau.

1. *Results according to the nature of the injury and mode of treatment.*

| | Cases. | Rec. | Died. | Cases. | Rec. | Died |
|---|---|---|---|---|---|---|
| *a. Fissure or fracture without primary affection of brain* | | | | 39 | 34 | 5 |
| No operation on the skull | 29 | 25 | 4 | | | |
| Removal of fragments | 8 | 7 | 1 | | | |
| Trephined | 2 | 2 | 0 | | | |
| | 39 | 34 | 5 | | | |
| *b. Fissure or fracture with primary affection of brain* | | | | 73 | 34 | 39 |
| No operation on the skull | 49 | 25 | 24 | | | |
| Removal of fragments | 4 | 3 | 1 | | | |
| Trephined | 20 | 6 | 14 | | | |
| | 73 | 34 | 39 | | | |
| *c. Fissure or fracture with depression, without primary affection of brain* | | | | 44 | 33 | 11 |
| No operation on the skull | 22 | 21 | 1 | | | |
| Removal of fragments | 9 | 6 | 3 | | | |
| Trephined | 13 | 6 | 7 | | | |
| | 44 | 33 | 11 | | | |
| *d. Fissure or fracture with depressiou, and with primary affection of brain* | | | | 145 | 88 | 57 |
| No operation on the skull | 50 | 39 | 11 | | | |
| Removal of fragments | 26 | 14 | 12 | | | |
| Trephined | 69 | 35 | 34 | | | |
| | 145 | 88 | 57 | | | |
| | | | | 301 | 189 | 112 |

| | | | |
|---|---|---|---|
| Thus there were treated without operation | 150 | 110 | 40 |
| " " by removal or elevation of fragments | 47 | 30 | 17 |
| " " by the trephine | 104 | 49 | 55 |
| | 301 | 189 | 112 |
| The trephine was employed prophylactically in | 16 | 15 | 1 |
| " therapeutically | 88 | 34 | 54 |
| " therapeutically in affection of brain without wounds | 1 | 0 | 1 |
| " therapeutically in affection of brain with wounds | 8 | 3 | 5 |
| | 113 | 52 | 61 |

Dr. Fritze compares these results of treatment with those derived from the treatment of the cases collected by Blassius and Leisnig. From the comparison, it appears that a greater proportion of recoveries upon the whole occurred in his series of cases than in theirs; but that the results in those cases where the trephine was resorted to, were much more favorable in their cases than in his. This he explains by the fact of all his fatal cases having been the subjects of medico-legal investigation, the whole number that occurred being declared, which was probably not the case with theirs. An impartial and searching criticism of the 112 fatal cases has led the author to the conviction, that, in only 10 of the number, it was possible that trephining, or the earlier resort to this, might have preserved life. But, on the other hand, in 2 of the fatal cases, the operation seemed to be the cause of death; while in 5 of the recoveries it was probably unnecessarily resorted to. The following is the comparative view of the cases:—

| | Cases. | Recoveries. | Per cent. |
|---|---|---|---|
| BLASIUS. | | | |
| Without operation | 242 | 83 | 34.3 |
| Trephine | 422 | 270 | 64 |
| | 664 | 353 | 53.2 |
| LEISNIG. | | | |
| Without operation | 260 | 118 | 45.4 |
| Trephine or removal of fragments | 223 | 173 | 77.6 |
| | 483 | 291 | 60 |
| FRITZE. | | | |
| Total cases | 301 | 189 | 62 |
| Without operation | 150 | 110 | 73.3 |
| Trephine | 104 | 49 | 47.1 |
| Trephine and removal of fragments | 151 | 79 | 52.3 |

2. *Results according to age.*

| | Cases. | Recoveries. | Died. | Per cent. of recoveries. |
|---|---|---|---|---|
| *Under* 15 *years of age.* | | | | |
| Without operation | 37 | 30 | 7 | 81 |
| Removal of fragments | 14 | 13 | 1 | 93 |
| Trephined | 18 | 11 | 7 | 61 |
| | 69 | 54 | 15 | |
| *Adults.* | | | | |
| Without operation | 113 | 79 | 34 | 70 |
| Removal of fragments | 33 | 17 | 16 | 51 |
| Trephined | 86 | 38 | 48 | 44 |
| | 232 | 134 | 98 | |

Thus the injuries proved less dangerous to the young, and operative interference was seldomer required; but when resorted to, the results were more favorable.

3. *Results according to sex.*

| | Cases. | Rec. | Died. | Per cent. of rec. |
|---|---|---|---|---|
| Men | 274 | 174 | 100 | 63.5 |
| Women | 26 | 13 | 13 | 50 |

4. *Results according to the seat of injury.* A statistical examination of 291 of the cases leads to the result that the minimum of danger exists when the frontal region is the seat of injury; then the vertex; and next the occiput. The danger is the greater, the more extensive the injury is, and the more it traverses the lateral portions of the cranium, towards the basis.

5. *Results according to the cause of the injury.* From an analysis of 298 cases, in which the nature of the injury is stated, it results that it arose from a blunt instrument in 121, from a fall in 100, from a stone in 31, from a pointed instrument in 12, from a kick of a horse in 12, from a cutting instrument in 13, and from firearms in 9. The following is the proportion in which these different injuries were recovered from, in relatiou to operative interference:—

| | Treated without operation. | Treated by operation. |
|---|---|---|
| Cutting instruments | 1.1 | 1 |
| Pointed " | 1 | 2.3 |
| Blunt " | 1.5 | 1 |
| Stone | 1.3 | 1 |
| Falls | 1.5 | 1 |
| Kicks | 1 | 1 |
| Firearms | 5 | 0 |

Statistics thus confirm what theory would teach us—that the operation of the trephine is successful in proportion as the cause of injury more immediately limits its operation to the part of the skull that is struck, which is most so the case in wounds from pointed bodies, and least so in those from firearms discharged close to the head.—*Brit. and For. Medico-Chirurg. Review, Jan.*, 1850, from *Casper's Wochenschrift, No.* 30.

